# Salicylic Acid Induction of Flavonoid Biosynthesis Pathways in Wheat Varies by Treatment

**DOI:** 10.3389/fpls.2016.01447

**Published:** 2016-09-28

**Authors:** Orsolya K. Gondor, Tibor Janda, Vilmos Soós, Magda Pál, Imre Majláth, Malay K. Adak, Ervin Balázs, Gabriella Szalai

**Affiliations:** ^1^Agricultural Institute, Centre for Agricultural Research, Hungarian Academy of SciencesMartonvásár, Hungary; ^2^Department of Botany, University of KalyaniKalyani, India

**Keywords:** flavonoids, gene expression, hydroponic treatment, salicylic acid, seed soaking, wheat

## Abstract

Salicylic acid is a promising compound for the reduction of stress sensitivity in plants. Although several biochemical and physiological changes have been described in plants treated with salicylic acid, the mode of action of the various treatments has not yet been clarified. The present work reports a detailed comparative study on the effects of different modes of salicylic acid application at the physiological, metabolomic, and transcriptomic levels. Seed soaking and hydroponic treatments were found to induce various changes in the protective mechanisms of wheat plants. The possible involvement of the flavonoid metabolism in salicylic acid-related stress signaling was also demonstrated. Different salicylic acid treatments were shown to induce different physiological and biochemical processes, with varying responses in the leaves and roots. Hydroponic treatment enhanced the level of oxidative stress, the expression of genes involved in the flavonoid metabolism and the amount of non-enzymatic antioxidant compounds, namely ortho-hydroxycinnamic acid and the flavonol quercetin in the leaves, while it decreased the ortho-hydroxycinnamic acid and flavonol contents and enhanced ascorbate peroxidase activity in the roots. In contrast, seed soaking only elevated the gene expression level of phenylalanine ammonia lyase in the roots and caused a slight increase in the amount of flavonols. These results draw attention to the fact that the effects of exogenous salicylic acid application cannot be generalized in different experimental systems and that the flavonoid metabolism may be an important part of the action mechanisms induced by salicylic acid.

## Introduction

Salicylic acid plays a role in several processes such as plant growth regulation, development and responses to biotic and abiotic stresses as a signal molecule in the induction of acclimation mechanisms (Khan et al., 2015). Plants often respond to environmental stresses with an increase in the endogenous SA level (Janda et al., 2014). SA is a promising compound for the reduction of stress sensitivity in the practical agriculture (Horváth et al., 2007). Several methods of SA application have been used to protect plants against various stresses, including soaking seeds in SA solution before sowing (Krantev et al., 2008; Popova et al., 2009; Agami and Mohamed, 2013; Belkadhi et al., 2015; Rehman et al., 2015), spraying or irrigating plants with SA (Ghazijahani et al., 2014; Jesus et al., 2015; Huang et al., 2016), or adding it to the growth medium of hydroponically grown plants (Marcińska et al., 2013; Horváth et al., 2015). However, although various biochemical and physiological changes have been described in SA-treated plants, the mode of action of the various treatments has not yet been clarified. Furthermore, although several studies have described the role of exogenous SA in stress adaptation processes, the effect of SA is somewhat ambiguous. It has not been proved whether changes in the plant SA level or in stress response mechanisms are caused by SA itself or by other components activated by SA. For example, in pea seeds, which were pre-soaked in SA solution the majority of the exogenous SA was bound in conjugated forms; and a *de novo* SA synthesis was occurred in other plant organs (Szalai et al., 2011). SA does not only induce direct protective mechanisms, but it may also cause oxidative stress in plants through the generation of ROS, thus participating in the development of stress symptoms (Borsani et al., 2001).

**FIGURE 1:**
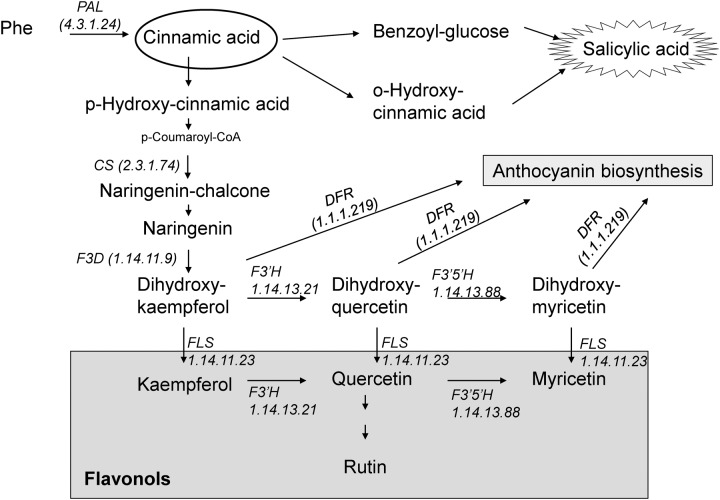
**Schematic presentation of salicylic acid and flavonoid biosynthesis.** (F3D, flavanone 3-hydroxylase; DFR, dihydroflavonol-4-reductase; CS, chalcone synthase; PAL, phenylalanine ammonia-lyase; FLS, flavonol synthase; F3′H, flavonoid 3′-hydroxylase; F3′5′H, flavonoid 3′,5′-hydroxylase).

Flavonoids are among the most bioactive secondary metabolites in plants. They constitute a secondary antioxidant system that is activated as a consequence of the depletion of antioxidant enzyme activity (Agati et al., 2012). They can serve as scavengers of ROS by locating and neutralizing radicals before they damage the cells and are thus important for plants under adverse environmental conditions (Løvdal et al., 2010). A considerable increase in flavonoid levels has been found following biotic and abiotic stresses, such as wounding, drought, metal toxicity, and nutrient deprivation (Gill and Tuteja, 2010; Agati et al., 2012). Many flavonoid biosynthesis genes are also induced under stress conditions. In general, stress-sensitive species display a less effective first line of defense against ROS under stressful conditions and are subsequently exposed to more severe oxidative stress. Therefore, the biosynthesis of flavonoids is often stimulated to a greater extent in stress-sensitive species than in stress-tolerant ones (Agati et al., 2012). It has been also suggested that flavonoids can be a secondary ROS scavenging system in the plants especially in photosynthetic tissues (Agati et al., 2012). Both SA and flavonoids are phenylpropanoids, which group has antioxidant activity, and are synthesized from phenylalanine *via* CA, an intermediate in the shikimic acid pathway (**Figure 1**). Flavonols represent a subgroup of flavonoids and are primarily synthesized from dihydroflavonols by flavonol synthase (FLS; Martens et al., 2010) (**Figure 1**). Flavonols were reported to play a more important role than xanthophylls in protecting *Arabidopsis* leaves from long-term visible light-induced oxidative damage (Havaux and Kloppstech, 2001). It has been also described that flavonol accumulation can increase abiotic stress tolerance better than other kind of phenolic compounds in tomato plants (Martinez et al., 2016).

The modes of action of SA as a protective compound are usually discussed very generally, irrespectively of the origin of the SA (endogenous synthesis or various types of exogenous application). The present work reports a detailed comparative study on the effects of different modes of SA applications – such as soaking of seeds in SA-solution before sowing and adding SA to the hydroponic solution – at the physiological, metabolomic, and transcriptomic levels in wheat. In the present paper it is shown that seed soaking and hydroponic treatment may induce different changes in the protective mechanisms. The involvement of the flavonoid metabolism in SA-related processes was also demonstrated.

## Materials and Methods

### Plant Material

Wheat seeds (*Triticum aestivum* L. variety Mv Emese) were soaked overnight either in distilled water (control plants) or in 0.5 mM SA (SA seed-soaked; SA-ss plants). The seeds were then germinated for 3 days at 22°C, after which the seedlings were grown in modified Hoagland solution (Pál et al., 2005) for 2 weeks at 20/18°C with 16/8-h light/dark periodicity and photosynthetic photon flux density (PPFD) of 250 μmol m^-2^ s^-1^in a Conviron G-48 plant growth chamber (Controlled Environments Ltd, Winnipeg, MB, Canada) in the phytotron of the Agricultural Institute, Centre for Agricultural Research, Hungarian Academy of Sciences, Martonvásár, Hungary. At the end of this period 0.5 mM SA was added to the hydroponic solution of some of the control plants for 1 days, after which the solution was replaced with control solution (SA-h plants). Leaf and root samples were collected 1 and 7 days after the 1-day hydroponic SA treatment.

### Chlorophyll-*a* Fluorescence Induction Measurement

The quantum yield of Photosystem II (PSII), indicated by the ΔF/Fm′ [(Fm′- Fs)/Fm′ chlorophyll fluorescence induction parameter, where Fm′ and Fs represent the maximum and steady-state chlorophyll fluorescence levels in the light-adapted state, respectively, was measured on fully expanded leaves using a pulse amplitude modulated fluorometer (PAM-2000, Walz, Effeltrich, Germany) as described by Janda et al. (1994).

### Estimation of Lipid Peroxidation

The lipid peroxidation analysis was based on the measurement of MDA level. After grinding 0.2 g of tissue in 600 μl 0.1% (w/v) trichloroacetic acid, followed by centrifugation at 12 000 *g* for 10 min, 300 μl of the supernatant was mixed with 2 ml of 0.5% (w/v) thiobarbituric acid in 20% (w/v) trichloroacetic acid and incubated at 90°C for 30 min. The MDA equivalent component levels were measured spectrophotometrically at 532 nm, with the subtraction of non-specific absorption at 600 nm.

The concentration of lipid peroxides, together with the oxidatively modified proteins, was then quantified in terms of the MDA level using an extinction coefficient of 155 mM^-1^ cm^-1^, and expressed as nM g^-1^ fresh weight (Thomas et al., 2004).

### Antioxidant Enzyme Assays

For the analysis of antioxidant enzyme activity, 0.5 g of tissue from the third leaves or the roots were homogenized in 2.5 mL of ice-cold Tris buffer (0.5 M, pH 7.5) containing 3 mM MgCl_2_ and 1 mM EDTA.

The catalase (CAT; EC 1.11.1.6) and ascorbate peroxidase (APX; EC 1.11.1.11) activity was measured as described previously (Pál et al., 2005).

The guaiacol peroxidase (POD; EC 1.11.1.7) activity was measured at 470 nm as described by Ádám et al. (1995). The reaction mixture consisted of 88 mM Na-acetate buffer (pH 5.5), 0.88 mM guaiacol, 0.0375% H_2_O_2_, and enzyme extract.

The glutathione reductase (GR; EC 1.6.4.2) activity was determined at 412 nm according to Smith et al. (1988). The reaction mixture contained 75 mM Na-phosphate buffer (pH 7.5), 0.15 mM diethylenetriamine-pentaacetic acid, 0.75 mM 5,5′-dithiobis (2-nitrobenzoic acid), 0.1 mM NADPH, 0.5 mM oxidized glutathione, and 50 ml plant extract in a total volume of 1 ml.

The glutathione-*S*-transferase (GST; EC 2.5.1.18) activity was measured spectrophotometrically at 340 nm (Mannervik and Guthenberg, 1981). The reaction mixture contained 72.7 mM Na-phosphate buffer (pH 6.5), 3.6 mM reduced glutathione, 1 mM 1-chloro-2,4-dinitrobenzene, and enzyme extract.

The activities were expressed in nkatal g^-1^ protein.

### Extraction of Salicylic Acid and Flavonols; Analytical Procedure

Flavonoids, SA and its precursors were measured according to Meuwly and Métraux (1993) and Pál et al. (2005) using 1 g plant material. Just prior to the HPLC analysis, the evaporated samples were resuspended in 500 μl of the HPLC starting mobile phase.

Salicylic acid and oHCA were quantified fluorimetrically (W474 scanning fluorescence detector, Waters, USA), with excitation at 317 nm and emission at 436 nm for oHCA, followed by excitation at 305 nm and emission at 407 nm for SA. The determination of BA, CA, and flavonols, namely rutin, myricetin, quercetin, and kaempferol, was performed by means of UV spectrophotometry in the range of 230–300 nm (W996 photodiode array detector, Waters, USA).

### Microarray Analysis

For the microarray experiment, three biological replicates were harvested and three technical replicates were isolated from each sample (each consisted of seven plants). RNA was isolated using an RNEasy Plant Mini Kit (Qiagen) and the samples were treated with DNase I (Qiagen) according to the manufacturer’s instructions. The RNA Integrity Number (RIN) of the samples was determined with an Agilent BioAnalyzer. After assessing the RNA quality, equal amount of RNA samples with RIN > 8 were pooled and used for cRNA amplification. The RNA amplification and labeling procedure were accomplished according to the manufacturer’s recommendations (Agilent). The cRNA of three biological replicates labeled with biotin were hybridized to the Agilent 4X44K Wheat Chip. The cRNA samples from the control, SA-ss and SA-h treatments were compared to each other in a simple loop design.

### Gene Expression and KEGG Analysis

For the microarray validation and real-time PCR expression analysis of SA-h and SA-ss responsive genes, samples were harvested using the same experimental design as for microarray analysis with three independent biological replicates. Reactions were performed in quadruplicate. RNA was isolated as described previously in the microarray section, and 1 μg RNA was reverse transcribed with the Quantitect Reverse Transcription Kit (Qiagen). The real-time PCR was performed with Applied Biosystems 7500 FAST using Fast SYBR Green detection chemistry (Life Technologies) and gene-specific primers. The ACTIN2 (TC234027) gene (forward: CCTTCAATGTTCCAGCCATGTA; reverse: ATAGTTGAGCCACCACTGAGCA) as the endogenous control was used. Confirmation of specific product amplification was achieved using PCR and T_m_ analysis. The PCR efficiency of the three primer pairs (derived from the log slope of the fluorescence versus cycle number in the exponential phase of each amplification plot) ranged from 95 to 98.5%. The relative ratio of threshold cycle (Ct) values between the endogenous control and the specific gene was calculated for each sample, together with their standard deviations. Based on the available annotation from rice orthologs, the up- or downregulated genes in a given experiment were assigned to the available Gene Ontology (GO) categories. GO analysis was performed with an agriGO toolkit using SEA (singular enrichment analysis), with Fisher’s test and Wheat Affymetrix Genome Array as a background (Zhou et al., 2010). Accessions assigned to the categories ‘phenylpropanoid pathway,’ ‘flavonol biosynthesis,’ and ‘flavonoid biosynthesis’ were selected and subjected to KEGG pathway analysis^1^.

### Principal Component Analysis (PCA)

The similarity of the gene expression data set of each comparison was analyzed on the basis of the variance–covariance of the log (Fold Change; logFC) values of the probe sequences using PCA (Hammer et al., 2001).

### Determination of Phenylalanine Ammonia Lyase (PAL) Activity

Phenylalanine ammonia lyase activity was measured according to Gao et al. (2008) using 1 g leaves and roots, and expressed as enzyme units per g fresh weight (U g^-1^FW).

### Statistical Analysis

The whole experimental design was repeated three times, and the representative set of experiments is shown. The microarray experiment was only carried out only once from the representative experiment. In each set of experiments the biochemical data and plant growth parameters represented the average of 5 and 20 measurements, respectively. The data were statistically evaluated using the standard deviation and *t*-test methods using the Microsoft Excel program.

## Results

### Physiological State of the Plants

Wheat plants were treated with 0.5 mM SA either hydroponically (SA-h) or by soaking seeds overnight (SA-ss). The hydroponic SA treatment significantly decreased the shoot and root length after 7 days (**Table 1**). Neither hydroponic treatment nor seed soaking caused significant changes in the quantum yield of PSII (Φ_PSII_; **Table 1**). The MDA level in the leaves substantially increased in the case of SA-h treatment (**Table 1**) and, interestingly, it was lower in the roots of SA-h plants than in the control after 7 days. SA-ss caused no change in the lipid peroxidation level either in leaves or roots.

**Table 1:** Changes in the physiological parameters of wheat plants after various exogenous SA treatments.

	Length (cm)		Quantum yield of PSII (Photosystem II)	MDA (nmol g^-1^ FW)	
			
	Shoot	Root		Leaf	Root
Control	23.3 ± 1.8	18.0 ± 2.8	0.672 ± 0.037	3.95 ± 0.50	1.65 ± 0.20
SA-ss	22.7 ± 1.6	19.9 ± 3.1^∗^	0.679 ± 0.001	3.62 ± 0.70	1.61 ± 0.30
SA-h	17.5 ± 1.9^∗∗∗^	8.4 ± 1.8^∗∗∗^	0.621 ± 0.049	15.2 ± 1.80^∗∗∗^	0.37 ± 0.00^∗∗^


*SA-ss, seed soaking in 0.5 mM SA prior to sowing; SA-h, 0.5 mM SA addition to the hydroponic solution for 1 days; ^∗^, ^∗∗^, ^∗∗∗^ significant differences compared to the control plants at the *p* < 0.05, 0.01, and 0.001 levels, respectively.*

### Antioxidant Enzyme Activity

Since SA-h treatment modified the MDA level, the activities of certain antioxidant enzymes were also determined. The antioxidant enzymes responded differently to the SA-h and SA-ss treatments (**Table 2**). The APX activity in the leaves substantially increased 7 days after the addition of SA to the hydroponic solution, and after 1 days in the roots. A decline in the GST activity was observed in both the roots and leaves of SA-h treated plants after 1 days, and in the CAT activity only in the leaves, while the POD activity in the roots was reduced after 7 days. In SA-ss plants, although the differences were never statistically significant in the roots, the enzyme activity differed slightly from that of the control plants in the leaves in certain phases of growth, suggesting that despite the lack of significant changes in the growth parameters or MDA level SA-ss treatment may have affected certain stress-related processes.

**Table 2:** Changes in the antioxidant enzyme activity in wheat plants after various exogenous SA treatments.

	Leaf						Root					
		
	1 days			7 days			1 days			7 days		
				
	Control	SA-ss	SA-h	Control	SA-ss	SA-h	Control	SA-ss	SA-h	Control	SA-ss	SA-h
CAT	1476 ± 176	1255 ± 120*	1209 ± 161*	1159 ± 160	1528 ± 309	1591 ± 310	33 ± 12	72 ± 30	30 ± 22	59 ± 29	61 ± 22	55 ± 26
POD	30.5 ± 6.4	35.7 ± 8.4	32.9 ± 6.0	72.7 ± 17.5	57.8 ± 15.3	92.8 ± 53.5	611 ± 181	492 ± 30	756 ± 77	752 ± 224	545 ± 56	510 ± 20*
GR	8.2 ± 1.0	6.8 ± 2.1	7.6 ± 0.5	8.0 ± 1.0	5.6 ± 0.5**	8.0 ± 2.0	12.8 ± 1.3	11.2 ± 1.5	9.4 ± 2.2	13.3 ± 4.4	7.6 ± 1.5	9.0 ± 1.1
APX	6.3 ± 1.5	7.5 ± 0.5	7.9 ± 1.8	6.5 ± 0.8	7.2 ± 0.4	11.3 ± 0.9***	31.3 ± 7.9	28.5 ± 16.6	117 ± 9***	38.9 ± 12.4	38.8 ± 11.8	75.4 ± 25.4*
GST	4.3 ± 0.5	3.5 ± 0.6*	3.4 ± 0.7**	4.4 ± 0.8	3.8 ± 0.6	3.6 ± 0.5	3.3 ± 0.7	4.1 ± 1.2	1.9 ± 0.5**	3.3 ± 1.1	2.6 ± 0.6	2.2 ± 0.8


*The activities were expressed in nkatal g^-1^ protein.*

*SA-ss, seed soaking in 0.5 mM SA prior to sowing; SA-h, 0.5 mM SA addition to the hydroponic solution for one day; CAT, catalase; POD, guaiacol peroxidase; GR, glutathione reductase; APX, ascorbate peroxidase; GST, glutathione-*S*-transferase; ^∗^, ^∗∗^, ^∗∗∗^ significant difference compared to the control plants at the *p* < 0.05, 0.01, and 0.001 levels, respectively.*

### *In vivo* Level of SA and Related Compounds

**FIGURE 2:**
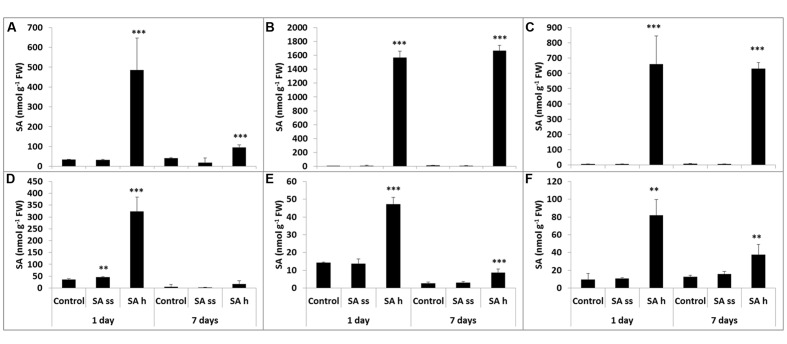
**Changes in the endogenous SA content after various exogenous SA treatments (SA-ss, seed soaking in 0.5 mM SA prior to sowing; SA-h, 0.5 mM SA addition to the hydroponic solution for 1 days) in the leaves **(A)** methanol-soluble free fraction; **(B)** methanol-soluble bound fraction; **(C)** methanol-insoluble bound fraction) and in the roots **(D)** methanol-soluble free fraction; **(E)** methanol-soluble bound fraction; **(F)** methanol-insoluble bound fraction.** ^∗∗^, ^∗∗∗^ significant differences compared to the control plants at the *p* < 0.01 and 0.001 levels, respectively.

The methanol-soluble free and bound and the methanol-insoluble bound fractions of SA, oHCA, BA, and CA were measured in the leaves and roots of SA-ss and SA-h plants. The SA-h treatment caused changes in the SA level both in the leaves and roots, but these were more pronounced in the leaves than in the roots in all the fractions. The SA content in the leaves increased after 1 days of SA-h treatment in all the fractions (**Figures 2A,B,C**). The free SA decreased after 7 days, though it was still higher than in the control plants (**Figure 2A**), while the amount of SA in the bound fractions remained at the 1-day level (**Figures 2B,C**). As in the leaves, the SA level in the roots increased after 1 days of SA-h treatment in all the fractions (**Figures 2D,E,F**) but a smaller amount of SA could be detected than in the leaves. After 7 days, however, the SA content decreased not only in the free but in the bound fractions too. SA-ss treatment caused no substantial difference in the SA level in either the leaves or the roots.

The free oHCA level was below the detection limit in the leaves of both control and SA-ss plants, but an increase could be detected after 1 days of SA-h treatment, which was more pronounced after 7 days (**Supplementary Table S1**). A small amount of oHCA could be observed in the control and SA-ss plants in the methanol-soluble bound fraction but the oHCA level of SA-h treated plants was below the detection limit. There was no detectable oHCA in the methanol-insoluble bound fraction either in the leaves or in the roots. oHCA could only be detected in the methanol-soluble bound fraction in the roots, and the quantity was about half of that in the leaves. It dropped below the detection limit after 1 days of SA-h treatment, and although a rise was observed after 7 days, it was still lower than the control values.

Benzoic acid was detected in the same order of magnitude in the leaves and roots. The free BA content rose after 1 days of SA-h treatment in the leaves, but it was below the detection limit after 7 days (**Supplementary Table S1**). In contrast after SA-h treatment BA decreased in the bound fraction after the 1 days and increased after 7 days. Changes were only detected in the methanol-insoluble bound fraction after 7 days: the BA level in the leaves increased in the SA-h treatment but decreased in the SA-ss plants. On the other hand, free BA was below the detection limit after SA-h treatment in the roots and similar changes were found in the methanol-soluble bound fraction after 1 days of SA-h treatment. Although it could be detected after 7 days the level was much lower than in the roots of control or SA-ss plants. The amount of methanol-insoluble bound BA was smaller in SA-ss treated roots than in the control at the first sampling data, but after that it started to increase.

A very small amount of free CA was found in the leaves. It increased after 1 days of SA-h treatment but dropped back to almost the initial level after 7 days (**Supplementary Table S1**). As in the case of BA, opposite changes were observed in the methanol-soluble bound form, which decreased after 1 days in SA-h treated leaves but significantly increased after 7 days. The levels of methanol-soluble bound CA in the leaves were much higher than that of the free form. The free and methanol-soluble bound fractions in the roots were of the same magnitude as the free CA fraction in the leaves. In the root the amount of free CA dropped below the detection limit in the SA-h treatment. Similar changes were observed in the methanol-soluble bound fraction after 1 days, but after 7 days it returned to the initial control value. No significant differences could be detected in the methanol-insoluble bound fraction.

### Transcriptome Analysis of SA-ss and SA-h Plants

In order to detect sets of genes whose expression was altered by different SA regimes, a microarray analysis was conducted. The transcriptomes of leaves of control, SA-ss and 1-day SA-h seedlings were compared to each other. Responsive transcripts were defined as those with mean signal intensities that increased or decreased in intensity (i.e., transcript abundance) at least twofold at a corrected *p*-value of <0.05 compared with the control (**Supplementary Table S2**). Using these criteria, large subsets of transcripts were identified which showed treatment-specific up- or down-regulation.

The scatter plot diagram of principle component analysis revealed similarity between the gene expression profiles of the control vs. SA-h, control vs. SA-ss, and SA-ss vs. SA-h comparisons (**Supplementary Figure S1**). The biplot representation showed that the control vs. SA-h and SA-ss vs. SA-h comparisons had very similar gene expression profiles, while the control vs. SA-ss comparison was less similar to the others. A greater number of outlier genes with different expression were distinguished along principal component 1, and these were responsible for the dissimilarity between control vs. SA-ss and the other comparisons. This also suggests that seed soaking and hydroponic treatment have different effects on the gene expression levels.

**FIGURE 3:**
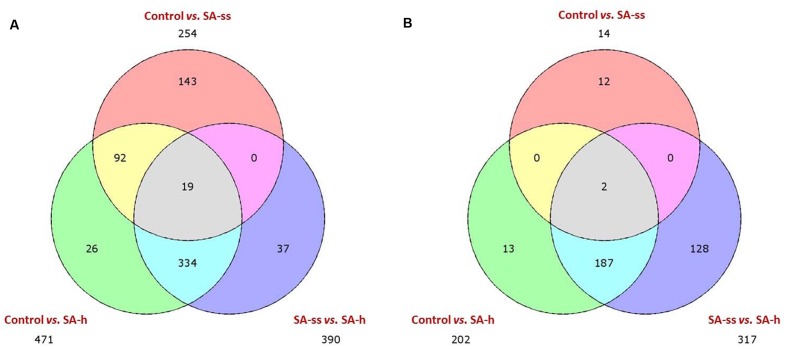
**Three-set Venn diagram comparisons of the annotated genes (*E*-value <1e–4).** The distribution of uniquely expressed and overlapping genes is shown on **(A)** (upregulated) and **(B)** (downregulated). (ctrl, control; SA-ss, seed soaking in 0.5 mM SA prior to sowing; SA-h, 0.5 mM SA addition to the hydroponic solution for 1 days).

The Venn diagram representing the number of differentially expressed genes in the different comparisons shows that SA-h treatment affected more genes than SA-ss treatment (**Figure 3**). Furthermore, both treatments mainly up-regulated the genes, and fewer genes were down-regulated compared to the control plants. Genes that were differentially expressed in the control vs. SA-h and control vs. SA-ss comparisons were enriched using GO annotation, and the distribution of the 40 distinct GO categories and the representativity of each category were analyzed (**Supplementary Figure S2**). This analysis confirmed that SA-h treatment up-regulated more genes than SA-ss. However, there were several genes which were differentially activated by SA-ss but not by SA-h. The modified genes belonged to a wide range of biochemical pathways, from primary metabolic processes to stress-related or regulating mechanisms.

**FIGURE 4:**
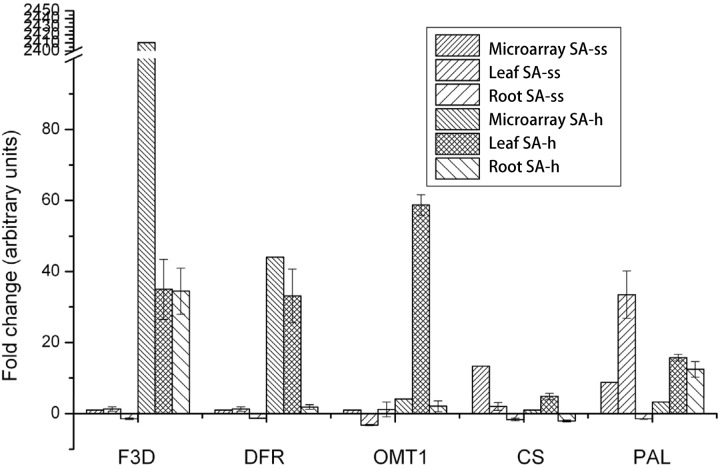
**Changes in the expression level of genes related to the flavonol metabolism in 1-week-old plants after various salicylic acid treatments.** (F3D, flavanone 3-hydroxylase; DFR, dihydroflavonol-4-reductase; OMT-1, flavone *O*-methyltransferase 1; CS, chalcone synthase; PAL, phenylalanine ammonia-lyase; ss, seed soaking in 0.5 mM SA prior to sowing; sh, 0.5 mM SA addition to the hydroponic solution for 1 days).

The control vs. SA-h and control vs. SA-ss comparisons revealed a huge shift in the expression of genes related to phenylpropanoid, flavonol, and flavonoid biosynthesis after both treatments. The genes F3D (flavanone 3-hydroxylase, 1.14.11.9, BAH36892.1), DFR (dihydroflavonol-4-reductase, 1.1.1.219, BAD11019.1), and OMT-1 (flavone *O*-methyltransferase 1, Q84N28.1) were considerably up-regulated in SA-h plants (**Figure 4**), while they showed little or no response in SA-ss plants, where only CS (chalcone synthase, 2.3.1.74, AHL27912.1) and PAL (phenylalanine ammonia-lyase, 4.3.1.24, EMT33318.1) were up-regulated (**Figure 4**). An independent real-time PCR experiment gave similar findings, thus validating the transcriptome results (**Figure 4**).

For characterization of the relatively early effects of seed soaking, the expression pattern of the above-mentioned genes was also assessed in the radicles and coleoptiles of 3-day-old SA-ss seedlings. Interestingly, CS and PAL showed a distinct down-regulation in the roots (**Supplementary Figure S3**). PAL was also repressed in the coleoptiles, while F3D was up-regulated. DFR was down-regulated in both tissues, and OMT-1 displayed no substantial change after the treatment. It should be noted that PAL was up-regulated in older plants, in contrast to the data obtained from fully developed, germinated seedlings.

### Flavonols

**FIGURE 5:**
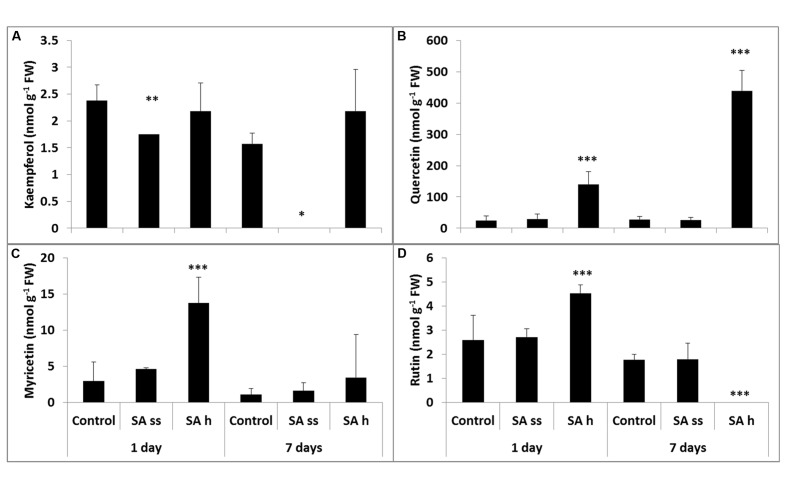
**Changes in the methanol-soluble free fraction of flavonols after various exogenous SA treatments in the leaves (SA-ss, seed soaking in 0.5 mM SA prior to sowing; SA-h, 0.5 mM SA addition to the hydroponic solution for 1 days; **(A)** kaempferol; **(B)** quercetin; **(C)** myricetin; **(D)** rutin).** ^∗^, ^∗∗^, ^∗∗∗^ significant difference compared to the control plants at the *p* < 0.05, 0.01, and 0.001 levels, respectively.

**FIGURE 6:**
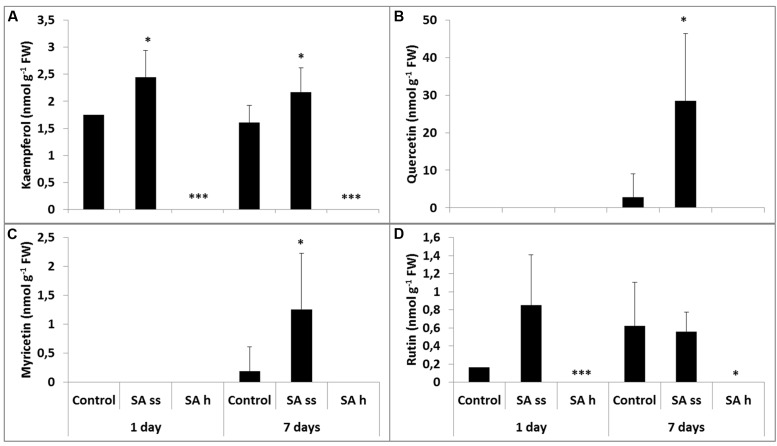
**Changes in the methanol-soluble free fraction of flavonols after various exogenous SA treatments in the roots (SA-ss, seed soaking in 0.5 mM SA prior to sowing; SA-h, 0.5 mM SA addition to the hydroponic solution for 1 days; **(A)** kaempferol; **(B)** quercetin; **(C)** myricetin; **(D)** rutin).** ^∗^, ^∗∗∗^ significant difference compared to the control plants at the *p* < 0.05 and 0.001 levels, respectively.

The total kaempferol contents (free and bound) were the lowest (**Figure 5A**), while quercetin could be found in the highest concentration both in the leaves and roots (**Figure 5B**). Soaking seeds in SA resulted in a decrease in the free kaempferol level in the leaves, but increased it in the roots. This treatment did not significantly modify the other flavonol compounds, except for a substantial increase in the quercetin level in the roots (free and bound forms; **Figure 6B**; **Supplementary Table S3**, respectively). The free quercetin content also increased four times after 1 days and twelve times after 7 days in the leaves of SA-h plants (**Figure 5B**). In contrast, a temporary decrease in the methanol-soluble bound fraction was found after 1 days, but a five times higher amount was detected after 7 days compared to the control plants (**Supplementary Table S3**). Similar changes were found in the free fractions of myricetin and rutin: while their concentrations increased after 1 days in the leaves of SA-h plants, they returned to the initial level or to an even lower value in the case of rutin after 7 days (**Figures 5C,D**). The methanol-soluble bound fraction also decreased at first, but dramatically increased after 7 days (**Supplementary Table S3**).

Changes in the roots were mainly detected in the free fraction. While the free kaempferol level was below the detection limit in SA-h plants, it was higher in the SA-ss plants than in the control (**Figure 6A**). Similar changes were found in the methanol-soluble bound fraction in the SA-h plants after 1 days, but there was no difference between the control and the treated plants in the kaempferol level of the roots after a further 6 days without SA (**Supplementary Table S3**). The free quercetin (**Figure 6B**) and myricetin (**Figure 6C**) contents were below the detection limit in the 8-day-old plants in every treatment; however, a slight increase was later observed in the SA-ss plants (**Figures 6B,C**). The level of rutin changed similarly in the roots as kaempferol content (**Figure 6D**).

**FIGURE 7:**
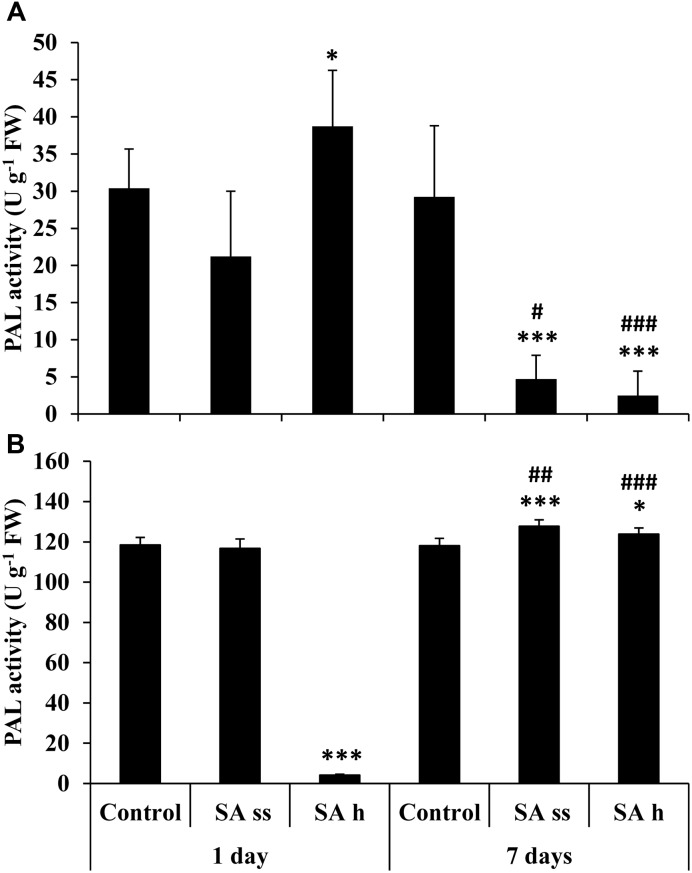
**Changes in the PAL activity after various exogenous SA treatments (SA-ss: seed soaking in 0.5 mM SA prior to sowing; SA-h: 0.5 mM SA addition to the hydroponic solution for 1 days) in the leaves **(A)** and roots **(B)** of wheat plants.** ^∗^, ^∗∗∗^ significant differences compared to the control plants at the *p* < 0.05 and 0.001 levels, respectively. **^#^**, **^##^**, **^###^** significant differences compared to plants given the same treatment 7 days earlier at the *p* < 0.05, 0.01, and 0.001 levels, respectively.

Phenylalanine ammonia lyase activity increased in the leaves of SA-h plants (**Figure 7A**) but decreased in the roots after 1 days (**Figure 7B**). In contrast, there was a dramatic decrease in the activity in the leaves after 7 days in both treatments, while a slight but significant increase was detected in the roots.

## Discussion

The exogenous application of SA has been shown to protect plants against various types of abiotic stress (Horváth et al., 2007). A whole-genome approach to determine the genes specifically regulated in response to exogenous SA has been used in the roots of a dicot plant, *Arabidopsis* (Badri et al., 2008). In the present work the physiological effects of two different modes of application, soaking seeds prior to sowing and adding SA to the hydroponic solution, were compared in the monocot wheat plants with high economic value. Our microarray study focused on the events in the leaves; and based on these results we completed it with comparative gene expression and biochemical analyses of leaves and roots. SA was applied at a concentration of 0.5 mM, because various studies described the positive effect of SA in this concentration range (for example, Janda et al., 1999; Krantev et al., 2008). Although a relatively large number of studies have described the role of SA mainly exogenously applied in stress adaptation processes, SA may also cause oxidative stress to plants, partly through the accumulation of hydrogen peroxide (Horváth et al., 2007). A larger quantity of ROS is usually generated in the leaves, as they are produced during photosynthesis. In the present experiment, the MDA content only increased in the leaves of SA-h plants, but since there was no substantial decrease in the quantum yield of PSII, this treatment did not cause serious damage to the plants. Alterations in the antioxidant activities of seed-soaked plants have also been reported in maize and pea (Krantev et al., 2008; Popova et al., 2009). Although SA-ss wheat plants exhibited no stress symptoms, this treatment also altered the activity of certain antioxidant enzymes.

The detailed microarray gene expression analysis indicated that especially the SA-h – and although to a lesser extent, the SA-ss treatment as well – differentially activated a large number of genes (**Supplementary Figure S2**). Majority of these genes are related to basic cellular/metabolic processes. The other main group of genes is related to responses of environmental stimuli or stress conditions. Interestingly, in contrast to SA-h, certain GO subcategories, especially localization, transport, developmental processes, or reproduction were not represented in the SA-ss plants.

Several of the differentially expressed genes, especially in the SA-h plants, are directly or indirectly related to the phenylpropanoid metabolism. Although earlier it was found by Badri et al. (2008) that treatment with SA caused significant changes in the phenylpropanoid biosynthesis in the roots of *Arabidopsis* plants but the flavonol metabolism was not investigated. Therefore, in the present study this secondary metabolic pathway was characterized in detail. It has been hypothesized that changes in the cellular redox homeostasis activate the biosynthesis of flavonoids, particularly the flavonol metabolism (Taylor and Grotewold, 2005). It was recently proved that an accumulation of flavonols over other kinds of phenolic compounds may contribute to the heat stress tolerance in tomato plants (Martinez et al., 2016). In the present work a substantial increase in the free quercetin fraction was detected in the leaves of SA-h plants, and this remained after the removal of SA from the hydroponic solution. The quercetin content of the methanol-soluble bound fraction decreased after 1 days compared to the control. This may have been the source of the higher free quercetin content but later the amount of methanol-soluble bound quercetin rose as well as that of the free fractions, suggesting an increase in the synthesis of quercetin. Quercetin derivatives are more effective than monohydroxy B-ring flavonoids in performing multiple functions in plants, which include the capacity to complex with Cu and Fe ions, thus inhibiting the generation of ROS in the Fenton reaction (Brown et al., 1998) as well as reducing ROS once formed. Quercetin derivatives may protect chloroplasts from the singlet oxygen generated by visible light. This hypothesis is consistent with the increase in a quercetin derivative, a dihydroxy B-ring-substituted flavonol, in preference to kaempferol, a monohydroxy B-ring flavonol, in response to white light irradiance. In contrast, the quercetin content of the roots was below the detection limit, while the kaempferol content was very low but detectable in the control and SA-ss plants. As leaves are exposed to light, quercetin was accumulated, especially in the SA-h treatment, while in the roots kaempferol could only be detected in the control and SA-ss plants.

Plants synthesize flavonols from CA, and this process has the same intermediate products as SA biosynthesis. The free CA level was low in the roots and decreased to below the detection limit after hydroponic SA treatment. Similar changes were found for the precursors (BA and oHCA) of SA, although the SA content increased in all three fractions. It can be assumed that the increase in the SA content in the roots originated mainly from the hydroponically added SA. In contrast to the roots, the free BA form increased in the leaves after 1 days, which could be responsible for the elevated SA level. In previous work using ^14^C-labeled SA radioactivity could not be detected in the increased SA content in the roots or leaves of pea plants. In the seeds radioactivity could only be detected in the bound form of SA, indicating that the absorbed SA was converted to methanol-soluble bound forms. The excess amount of free SA, which may be harmful to plants, thus appears to have been converted to the bound form (Szalai et al., 2011). The present work gave similar results: SA could be found mainly in bound forms in the leaves of hydroponically treated plants, but the amount of free SA in SA-h plants was almost the same as in the control after 7 days.

The level of oHCA, the other precursor of SA, increased to a much greater extent than BA in the leaves of SA-h treated plants and this increase was more pronounced after 7 days, when a higher MDA level was also detected. Hydroxycinnamic acids, a group of phenolics highly abundant in cereals, exhibit good antioxidant properties. Ferulic acid and its oxidative products, diferulic acids, are the most abundant hydroxycinnamic acids in cereals, but small quantities of other hydroxycinnamic acids (sinapic acid, oHCA, and caffeic acid) have also been described (Gallardo et al., 2006). As the antioxidant ability of hydroxycinnamic acids, including oHCA, is demonstrated by their ability to quench singlet molecular oxygen, it was suggested that an increase in the oHCA content could be induced independently of SA biosynthesis, and might play a role in the antioxidative response (Foley et al., 1999). A substantial increase in the bound oHCA level was also observed in winter wheat plants during both the cold hardening period (Janda et al., 2007) and salt stress (Szalai and Janda, 2009), so oHCA may not only serve as a precursor of SA, but may also have an antioxidant role during abiotic stress and recovery, and could also be involved in adaptation processes.

In summary, there was a rapid increase in PAL activity in the leaves of SA-h plants and this was confirmed by an increase in gene expression. This may serve as a stress signal, followed by an increase in SA and quercetin, parallel with the synthesis of myricetin and rutin. The removal of SA from the hydroponic culture caused the PAL activity and the levels of myricetin and rutin to return to the initial level. However, the level of bound SA remained high and there was a further increase in the quercetin level. This may be due to a long-term memory of plants (Crisp et al., 2016).

Lower levels of these compounds could be detected in the roots. SA-h treatment decreased the PAL enzyme activity despite a slight increase in the gene expression level, suggesting that PAL activity is regulated at the post-transcriptional level. The root SA content also increased, but in contrast to the leaves, both the free and bound forms decreased after the removal of SA from the hydroponic. The levels of the free flavonols decreased, but the accumulation of bound quercetin could be detected.

The effect of SA-ss was less pronounced, but a progressive decrease in PAL activity could be observed in the leaves. Similarly, the expression of the PAL gene was down-regulated in the young, 3-day-old plants. This decrease did not affect the SA level, but there was a decrease in the kaempferol content in the SA-ss leaves.

## Conclusion

The present results showed that different kinds of SA treatments induced different physiological and biochemical processes, and that the responses also differed in the leaves and roots. SA-h treatment elevated the level of oxidative stress in the leaves, induced an increase in the expression level of genes involved in the flavonoid metabolism and increased the amount of non-enzymatic antioxidant compounds, namely oHCA, a precursor of SA, and the flavonol quercetin. These changes may contribute to the enhanced stress tolerance induced by exogenous SA. However, it caused a decrease in the oHCA and flavonol contents, while enhancing the activity of APX in the roots. In contrast, SA-ss treatment only elevated the gene expression level of PAL in the roots and caused a slight increase in the amount of flavonols. These results draw attention to the fact that the effects of exogenous SA application cannot be generalized in different experimental systems and that the flavonoid metabolism may be an important part of the action mechanisms induced by SA.

## Author Contributions

GS and TJ designed the experiment; VS prepared samples for microarray, and together with IM analyzed the data. EB helped in data interpretation. MA and MP measured enzyme activities and helped in manuscript preparation. Biochemical and analytical measurements and data evaluations were carried out by OG and GS. GS and TJ prepared the manuscript. All authors read and approved the final manuscript.

## Conflict of Interest Statement

The authors declare that the research was conducted in the absence of any commercial or financial relationships that could be construed as a potential conflict of interest.

## Footnotes

1 http://www.genome.jp/kegg/pathway.html

## Abbreviations

APX: ascorbate peroxidase
BA: benzoic acid
CA: cinnamic acid
CAT: catalase
GR: glutathione reductase
GST: glutathione-*S*-transferase
MDA: malondialdehyde
oHCA: *ortho*-hydroxycinnamic acid
PAL: phenylalanine ammonia lyase
POD: guaiacol peroxidase
ROS: reactive oxygen species
SA: salicylic acid
