# Supplementary material for: Salicylic Acid Induction of Flavonoid Biosynthesis Pathways in Wheat Varies by Treatment
Source: Front Plant Sci. 2016 Sep 28;7:1447. doi: 10.3389/fpls.2016.01447 (PMC5039175; doi:10.3389/fpls.2016.01447)
Supplement: Supplementary file 1 [file Table_1.PDF]

Table S1. Changes in the methanol-soluble free and bound fractions of phenolic compounds related to the SA metabolism in wheat plants after various exogenous SA treatments.

		Leaf						Root					
		1 day			7 days			1 day			7 days		
		Control	SA-ss	SA-h	Control	SA-ss	SA-h	Control	SA-ss	SA-h	Control	SA-ss	SA-h
Cinnamic acid	Free	2.19 ± 1.69	0.54 ± 0.74	25.91 ± 25.22	0.81 ± 0.30	0.84 ± 0.34	4.72 ± 6.68	0.90 ± 0.39	0.81 ± 0.30	nd **	0.67 ± 0.00	0.40 ± 0.37	nd
	Bound	57.35 ± 11.44	41.30 ± 12.60	2.43 ± 0.37 ***	80.30 ± 14.63	75.84 ± 6.45	428.48 ± 22.90 ***	21.26 ± 0.48	14.71 ± 5.36	nd ***	9.72 ± 4.79	7.42 ± 1.72	24.29 ± 0.95 **
Benzoic acid	Free	43.24 ± 5.09	40.79 ± 5.69	82.88 ± 14.19 ***	22.44 ± 2.87	24.08 ± 15.75	nd ***	42.04 ± 4.12	38.00 ± 6.62	nd ***	29.81 ± 1.89	32.27 ± 9.87	nd ***
	Bound	75.76 ± 17.72	72.07 ± 11.60	1.80 ± 0.37 ***	142.10 ± 53.10	179.6 ± 11.80	349.7 ± 206.2	66.07 ± 3.78	70.43 ± 14.73	nd ***	92.22 ± 22.93	97.79 ± 11.56	20.20 ± 0.47 ***
o-Hydroxy-cinnamic acid	Free	nd	nd	312.3 ± 62.62 ***	nd	nd	936.7 ± 138.7 ***	nd	nd	nd	nd	nd	nd
	Bound	11.57 ± 5.17	13.40 ± 3.02	nd ***	20.58 ± 5.01	27.53 ± 6.90	nd ***	7.95 ± 0.04	6.29 ± 0.93	nd ***	6.09 ± 2.07	6.46 ± 2.09	1.42 ± 0.35 **

SA-ss: seed soaking in 0.5 mM SA prior to sowing; SA-h: 0.5 mM SA addition to the hydroponic solution for one day; *, **, *** significant differences compared to the control plants at the $p < 0.05$, 0.01 and 0.001 levels, respectively.
