# Supplementary material for: Salicylic Acid Induction of Flavonoid Biosynthesis Pathways in Wheat Varies by Treatment
Source: Front Plant Sci. 2016 Sep 28;7:1447. doi: 10.3389/fpls.2016.01447 (PMC5039175; doi:10.3389/fpls.2016.01447)
Supplement: Supplementary file 3 [file Table_3.PDF]

Table S3. Changes in the methanol-soluble bound fraction of flavonols in wheat plants after various exogenous SA treatments.

	Leaf						Root					
	1 day			7 days			1 day			7 days		
	Control	SA-ss	SA-h	Control	SA-ss	SA-h	Control	SA-ss	SA-h	Control	SA-ss	SA-h
Kaempferol	2.38 ± 0.29	1.82 ± 0.94	1.75 ± 0.00 ***	2.03 ± 1.22	2.17 ± 0.76	nd	1.75 ± 0.00	2.45 ± 0.49 **	nd ***	1.19 ± 0.88	2.03 ± 0.16	2.21 ± 1.01
Quercetin	229.4 ± 154.1	302.8 ± 210.4	2.98 ± 0.47 **	140.9 ± 96.97	183.6 ± 68.67	910.9 ± 47.26 ***	302.7 ± 220.4	477.9 ± 309.5	395.7 ± 132.9	417.5 ± 250.8	928.5 ± 178.2 *	1640.6 ± 974.0 *
Myricetin	18.92 ± 15.66	14.64 ± 12.37	0.13 ± 0.17 **	10.12 ± 6.16	12.57 ± 2.50	51.69 ± 1.56 c	4.29 ± 1.27	4.27 ± 1.23	7.70 ± 3.78	2.51 ± 0.77	1.96 ± 1.13	5.34 ± 4.25
Rutin	42.00 ± 32.47	36.30 ± 26.36	0.69 ± 0.07 **	49.14 ± 7.39	46.85 ± 2.76	112.5 ± 2.08 ***	3.00 ± 0.58	3.11 ± 1.71	0.98 ± 1.70	2.72 ± 1.10	2.49 ± 0.76	1.06 ± 0.12

SA-ss: seed soaking in 0.5 mM SA prior to sowing; SA-h: 0.5 mM SA addition to the hydroponic solution for one day; *, **, *** significant differences compared to the control plants at the $p < 0.05$, 0.01 and 0.001 levels, respectively.
