# Supplementary material for: Salicylic Acid Induction of Flavonoid Biosynthesis Pathways in Wheat Varies by Treatment
Source: Front Plant Sci. 2016 Sep 28;7:1447. doi: 10.3389/fpls.2016.01447 (PMC5039175; doi:10.3389/fpls.2016.01447)
Supplement: Supplementary file 5 [file Image_2.PDF]

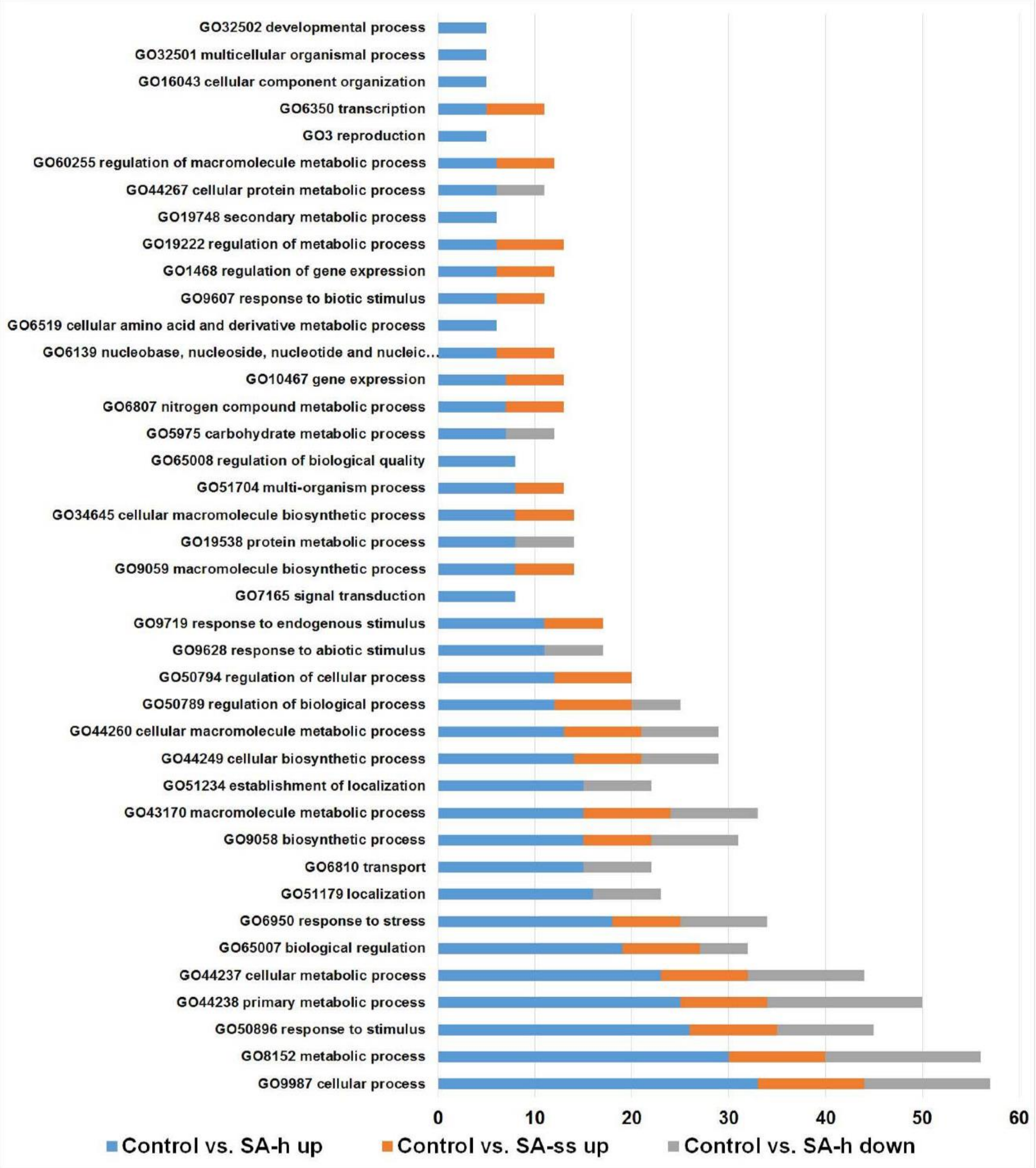

Figure S2. Figure S2. Distribution of gene ontology (GO) categories over different biological processes in genes which were differentially expressed in the control vs SA-h and control vs SA-ss comparisons. Up- and downregulated genes are presented separately. Downregulated genes in the control vs SA-ss comparison were not analyzed due to the low number of genes with known function and E-value < $1e^{-4}$ in this group. *X* and *y*-axes show the number of investigated genes in a group and the GO categories, respectively (SA-h: hydroponic treatment and SA-ss: seed soaking).
