# Supplementary material for: Salicylic Acid Induction of Flavonoid Biosynthesis Pathways in Wheat Varies by Treatment
Source: Front Plant Sci. 2016 Sep 28;7:1447. doi: 10.3389/fpls.2016.01447 (PMC5039175; doi:10.3389/fpls.2016.01447)
Supplement: Supplementary file 6 [file Image_3.PDF]

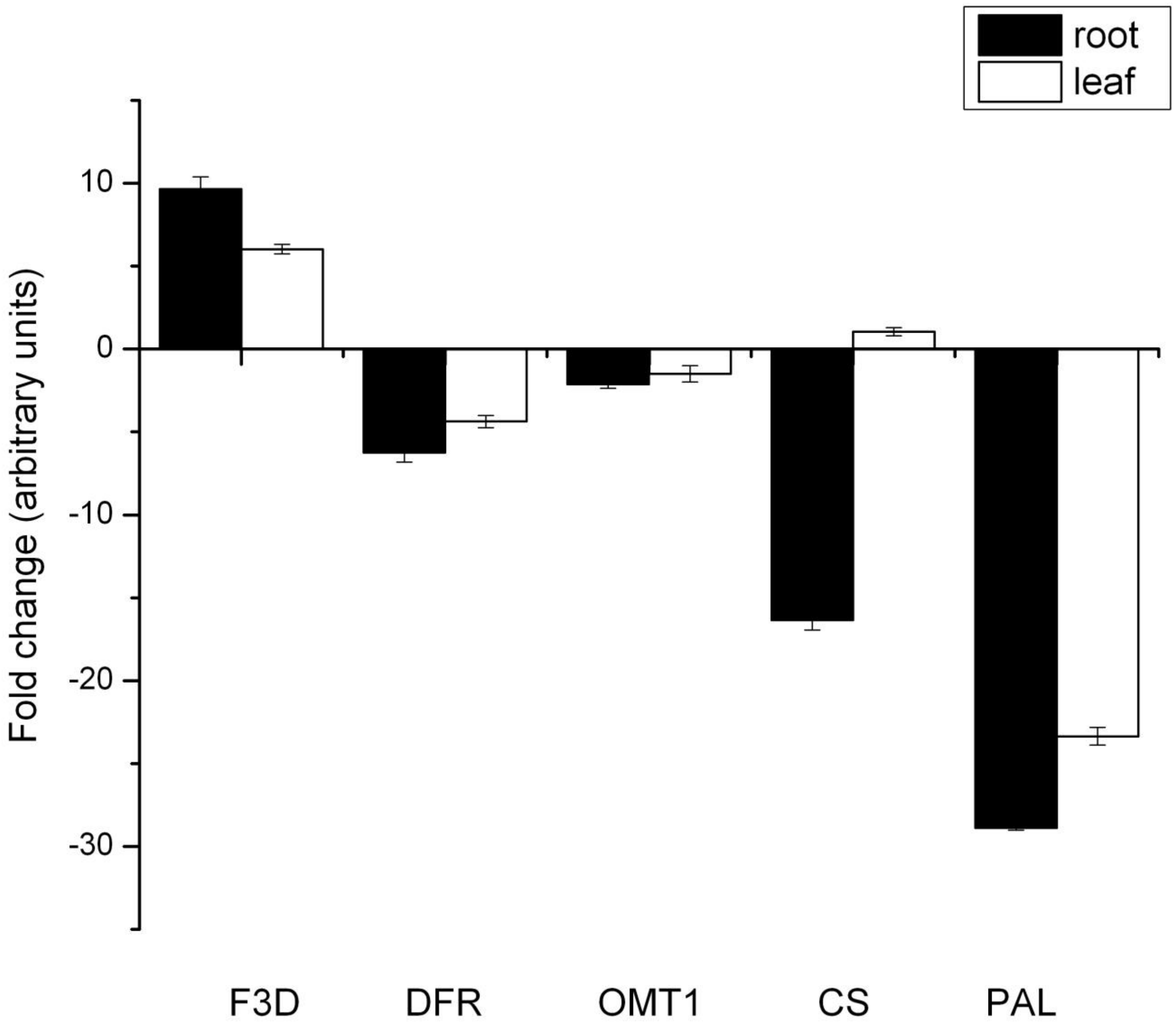

Figure S3. Changes in the expression level of genes related to the flavonol metabolism in three-day-old seedlings after seed soaking in 0.5 mM SA prior to sowing. (F3D: flavanone 3-hydroxylase; DFR: dihydroflavonol-4-reductase; OMT-1: flavone O-methyltransferase 1; CS: chalcone synthase; PAL: phenylalanine ammonia-lyase).
