# Supplementary material for: Salicylic Acid Induction of Flavonoid Biosynthesis Pathways in Wheat Varies by Treatment
Source: Front Plant Sci. 2016 Sep 28;7:1447. doi: 10.3389/fpls.2016.01447 (PMC5039175; doi:10.3389/fpls.2016.01447)
Supplement: Supplementary file 4 [file Image_1.PDF]

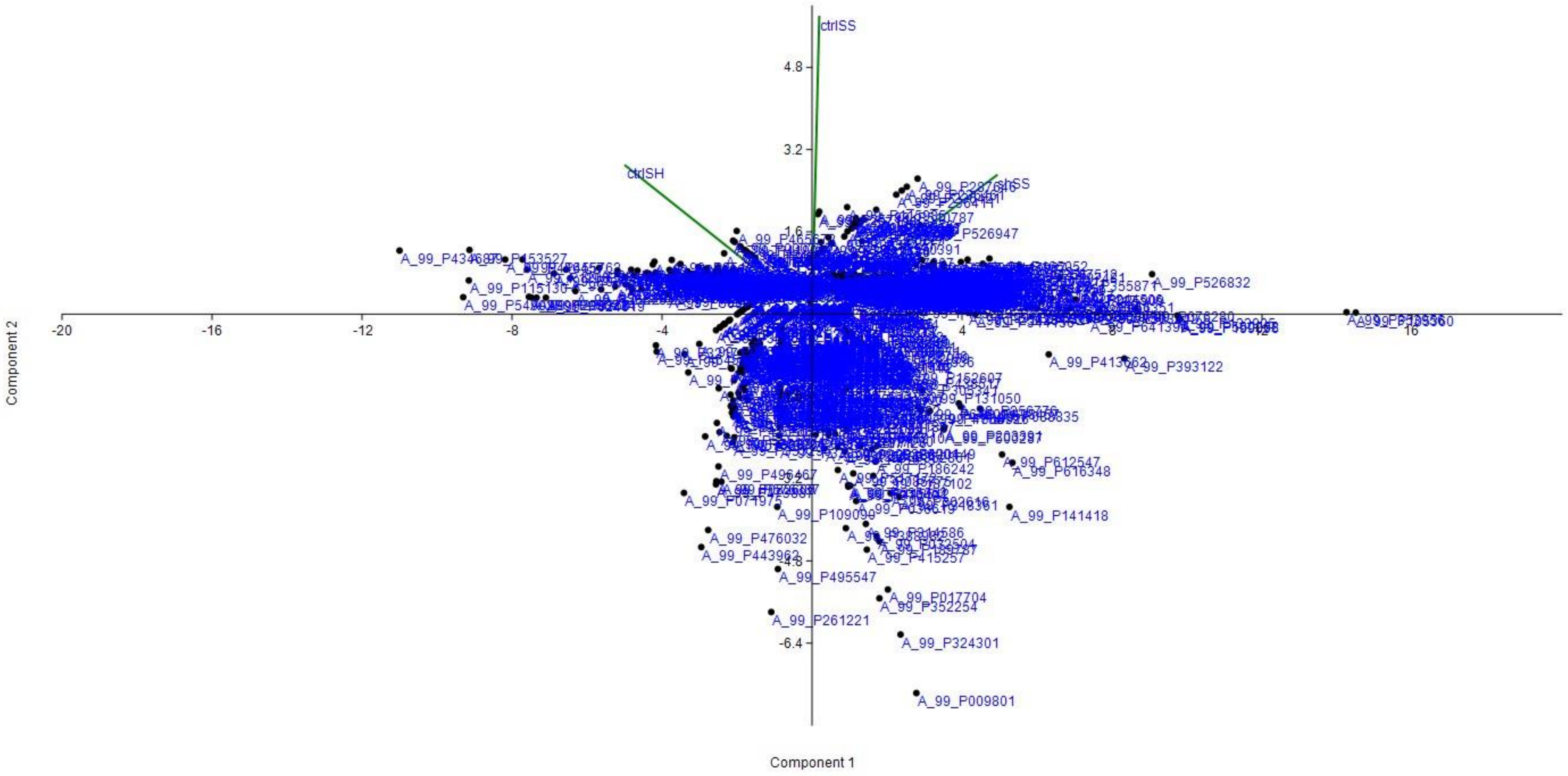

Figure S1. Scatter plot based on the logFC values of the gene expression patterns of all the comparisons. All the genes were plotted with respect to the first and second principal components and are represented in blue letters. The axes represent principal components 1 and 2, and the biplot (red lines) shows the projection of the original axes (treatments) onto the scatter diagram (in the comparisons, SA-ss: seed soaking and SA-h: hydroponic treatment).
